# Management of patients with persistent medically unexplained symptoms: a descriptive study

**DOI:** 10.1186/s12875-018-0791-9

**Published:** 2018-06-18

**Authors:** Kate Sitnikova, Rinske Pret-Oskam, Sandra M. A. Dijkstra-Kersten, Stephanie S. Leone, Harm W. J. van Marwijk, Henriëtte E. van der Horst, Johannes C. van der Wouden

**Affiliations:** 1 Department of General Practice and Elderly Care Medicine, Amsterdam Public Health Research Institute, VU University Medical Center, Van der Boechorststraat 7, 1081 BT Amsterdam, the Netherlands; 2 Department of Public Mental Health, Trimbos Institute: Netherlands Institute of Mental Health and Addiction, Da Costakade 45, 3521 VS Utrecht, the Netherlands; 3 Division of Primary Care and Public Health, Brighton and Sussex Medical School, Mayfield House, University of Brighton, Falmer, Brighton, BN1 9PH UK

**Keywords:** Disease management, General practice, Medically unexplained symptoms, Primary health care

## Abstract

**Background:**

In 2013 the Dutch guideline for management of medically unexplained symptoms (MUS) was published. The aim of this study is to assess medical care for patients with persistent MUS as recorded in their electronic medical records, to investigate if this is in line with the national guideline for persistent MUS and whether there are changes in care over time.

**Methods:**

We conducted an observational study of adult primary care patients with MUS. Routinely recorded health care data were extracted from electronic medical records of patients participating in an ongoing randomised controlled trial in 30 general practices in the Netherlands. Data on general practitioners’ (GPs’) management strategies during MUS consultations were collected in a 5-year period for each patient prior. Management strategies were categorised according to the options offered in the Dutch guideline. Changes in management over time were analysed.

**Results:**

Data were collected from 1035 MUS consultations (77 patients). Beside history-taking, the most frequently used diagnostic strategies were physical examination (24.5%) and additional investigations by the GP (11.1%). Frequently used therapeutic strategies were prescribing medication (24.6%) and providing explanations (11.2%). As MUS symptoms persisted, GPs adjusted medication, discussed progress and scheduled follow-up appointments more frequently. The least frequently used strategies were exploration of all complaint dimensions (i.e. somatic, cognitive, emotional, behavioural and social) (3.5%) and referral to a psychologist (0.5%) or psychiatrist (0.1%).

**Conclusions:**

Management of Dutch GPs is partly in line with the Dutch guideline. Medication was possibly prescribed more frequently than recommended, whereas exploration of all complaint dimensions, shared problem definition and referral to mental health care were used less.

## Background

Medically unexplained symptoms (MUS), i.e. physical symptoms that cannot entirely be accounted for by a known somatic disease, are extremely common in primary care [1, 2]. Although most such symptoms are self-limiting, in some cases they persist and impair patients’ functioning [3]. In the latter case, persisting MUS may meet diagnostic criteria for (undifferentiated) somatoform disorder of the psychiatric classification system DSM-IV [4]. Since the introduction of DSM-5, somatoform disorders have been replaced by somatic symptom disorders [5]. The main criteria for somatic symptom disorder no longer require the nature of physical symptoms to be unexplained, but focus on maladaptive cognitions, emotions and/or behaviour with respect to the physical symptom(s).

The prevalence of persistent MUS, such as those classified as somatoform disorders, is 3–10% in general practice [6–8]. Persistent MUS are disabling and are associated with high rates of comorbid mental health disorders [6, 9, 10]. There are high direct and indirect health care costs due to increased health care use and productivity loss due to sickness absence [11].

Previous research shows that GPs may view MUS patients as challenging, as it can be difficult for the GP to exclude the possibility of a serious illness and at the same time satisfy patients’ concerns about their health [12]. GPs’ may develop a sense of uncertainty in their professional knowledge [12–14] and patients may be left feeling that their symptoms are not being taken seriously [13].

To aid GPs in the management of patients with MUS, the Dutch College of General Practitioners published a guideline in 2013 [15]. Previous guidelines for MUS have also been published in Germany [16] and England [17]. The diagnostic recommendations in the Dutch guideline include ample exploration of all dimensions of complaints (i.e. somatic, cognitive, emotional, behavioural and social dimensions) and a thorough physical examination. The GP should be cautious with additional investigations and diagnostic referrals and should evaluate the severity of the symptoms or a change in symptoms over time. The therapeutic recommendations describe a stepped-care process in three steps, in which the GP starts with the mildest possible treatment and intensifies treatment when there are no adequate results.

It is unclear what current management for persisting MUS entails and whether this is in line with the Dutch guideline. Although GPs’ perceptions about giving explanations to patients with persistent MUS have previously been investigated in a Dutch focus group study, the actual management strategies were not described [18].

The aim of this descriptive study is to gain more insight into the management of adult patients with persistent MUS that meet criteria for an undifferentiated somatoform disorder in Dutch general practice and its potential change in time, as recorded in the patients’ medical records. We also aim to investigate to what extent this care is in line with the national guideline published by the Dutch College of General Practitioners.

## Method

### Study design and patient selection

We analysed the longitudinal electronic medical record data of persons participating in an ongoing randomised controlled trial (RCT) called the CIPRUS study. The CIPRUS study aims to establish the effectiveness of treatment of undifferentiated somatoform disorder by a mental health nurse practitioner (MHNP) within general practice, versus usual care. The design of the CIPRUS study has been described elsewhere in more detail [19]. Potential participants were identified by running a search of the electronic medical records for patients who had consulted their GP at least twice in the previous 3 months with one or more complaints from the Robbins list [20]. The Robbins list consists of 23 physical symptoms that are associated with functional somatic syndromes. GPs then checked the selected patients to verify that these patients indeed had MUS according to them, and excluded patients who fulfilled one or more exclusion criteria. Exclusion criteria were: presence of a medical or psychological disorder explaining the symptoms, presence of a severe psychiatric disorder, currently receiving psychological treatment for MUS, having poor language skills or handicap that prevented patients from understanding the intervention. After patients were verified as having MUS by their GP, they were interviewed using a structured clinical interview (SCID-I) in order to determine whether they fulfilled the DSM-IV criteria for undifferentiated somatoform disorder (USD) [21]. Those fulfilling the criteria for USD were included in the current study. All participating patients gave written informed consent to extract data from their electronic medical records. In the current study, we used data from patients participating in the usual care group of the CIPRUS study. We used data from the group of MUS patients receiving usual care because we wanted to know which care patients received. Because we had already identified these patients as having MUS we used the data from this group. Obviously we could not use data from the intervention group, as the intervention consisted of a number of scheduled meetings with the MHNP within the general practice, which would also be recorded in the electronic medical records. This data would, therefore, not only reflect usual care, but also care provided due to being part of the intervention group of our trial.

### Data extraction

Data were manually extracted by 3 researchers from electronic medical records of all participating patients between 21 November 2016 until 31 August 2017 in 30 participating general practices. Data were extracted for all MUS consultations in the 5-year time period for each patient prior to the search date. Data were collected from fields for prescription, medical tests and referrals, and from the GPs’ free text notes. Extracted data consisted of the date of the consultation, International Classification of Primary Care (ICPC) code corresponding to the consultation, and the management strategy of the GP, i.e. GPs’ own notes in the electronic medical records on what was carried out during the consultation and what they were planning or had arranged to do as a next step. For persons who were younger than 18 years of age during the 5-year time period, data were only collected from age 18 onward. MUS consultations were defined as consultations in which the GPs used ICPC codes that corresponded with the symptoms from the Robbins list (Table 1) [20]. Because there are no suitable corresponding ICPC codes for the symptoms ‘restlessness’ and ‘thoughts slower’, these two items from the Robbins list were omitted. Data from consultations that were not coded with ICPC codes corresponding to the Robbins list, but where the GP had noted ‘MUS’ or ‘somatisation’ were also collected.

**Table 1:** Robbins list and corresponding ICPC codes

Symptoms from the Robbins list	Corresponding ICPC codes	Number of consultations (%)
Back pain	L01, L02, L03	179 (17.3)
Joint pain	L20	28 (2.7)
Extremity pain	L18^a^	114 (11.0)
Headaches	N01, N02	69 (6.7)
Weakness/fatigue	A04	132 (12.8)
Chronic fatigue syndrome	A04.01	65 (6.3)
Sleep disturbance	P06^a^	106 (10.2)
Difficulty concentrating	P20	2 (0.2)
Loss of appetite	T03	0 (0.0)
Weight change	T07, T08	9 (0.9)
Restlessness	N/A	N/A
Thoughts slower	N/A	N/A
Chest pain	L04	38 (3.7)
Shortness of breath	R02	7 (0.7)
Palpitations	K04	24 (2.3)
Dizziness	N17^a^	29 (2.8)
Lump in throat	R21^a^	28 (2.7)
Numbness	N06^a^	5 (0.5)
Nausea	D09	16 (1.5)
Loose bowels	D11	20 (1.9)
Gas or bloating	D08	0 (0.0)
Constipation	D12	36 (3.5)
Abdominal pain	D01	57 (5.5)
Other (not part of the Robbins list)	A97, D93, P75, P78	71 (6.9)

^a^including subcodes

*N/A* not applicable

### Data categorisation

After collection, the extracted data on management were categorised by one researcher (KS) according to the options for diagnosis and treatment in the current Dutch GP guideline [15]. The categories from the guideline used for classifying diagnostic strategies were exploration of all complaint dimensions, physical examination and additional diagnostic testing within and outside general practice (diagnostic referral). The categories used for classifying treatment strategies were shared problem definition, education and explanation, advice, treatment with medication, setting up a time contingent plan, scheduling follow-up appointments, referral to other primary care providers, and referral to secondary care [15]. Within primary care a patient can be referred to other care providers such as a (psychosomatic) physiotherapist or exercise therapist, mental health nurse practitioner, primary care social psychiatric nurse or primary care psychologist (e.g. trained in cognitive behavioural therapy) [15].

### Data analysis

Data were analysed using SPSS version 22 for Windows. We used descriptive statistics to describe the study population and the management strategies. In order to determine whether there were any trends of providing various management strategies over time, we used cross-tabs and the chi-square test for trend.

## Results

**Fig. 1:**
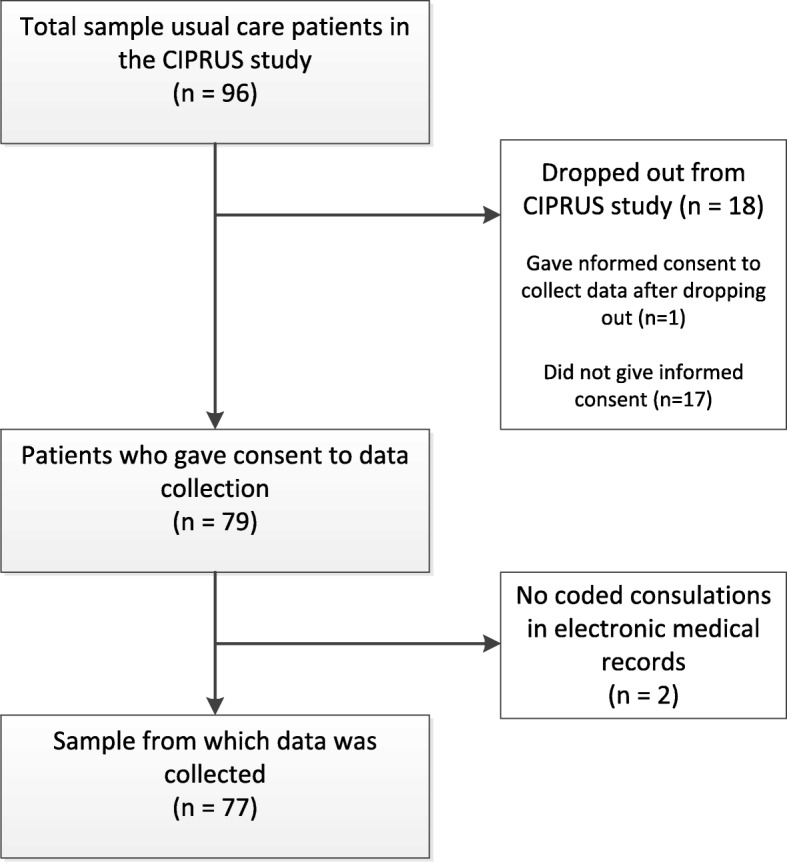
Flow chart of MUS patients. CIPRUS study = Cognitive-behavioural Intervention for PRimary care patients with Undifferentiated Somatoform disorder

Figure 1 presents a flow chart of patients included in this study. The control group of the CIPRUS study consisted of 96 patients in total. Seventeen patients dropped out of the study, and did not give permission to collect data from their medical records. Therefore, data were collected from 79 patients. For two patients, no information on MUS consultations was found in their electronic medical records. Therefore, data from 77 patients were available. The GPs registered a total of 1035 MUS consultations for these patients, of which 13.6% took place before 2013, the year in which the Dutch GP guideline was published.

### Characteristics of patients with persistent MUS

Of the 77 patients, 80.5% were female. The mean age was 50 (SD: 17.1, range: 19–89). Over the 5 year period, the mean number of MUS consultations was 13 (SD: 17, range: 1–130), resulting in an average of 2.6 consultations a year. The symptoms patients presented with are provided in Table 1. The most frequently recorded symptoms were back pain (17,3%), weakness or fatigue (12,8%), extremity pain (11%) and sleep disturbance (10,2%). No consultations had codes for loss of appetite and gas or bloating. Seventy-one consultations (6,9%) were coded with codes other than those that appear on the Robbins list, however the GP had referred to MUS in these consultations. The codes used in this category were hysteria/hypochondria (ICPC code P75), neurasthenia/stress (P78), spastic colon/irritable bowel syndrome (D93), and ‘no disease’ (A97).

### Recorded management strategies

GPs varied in the way they recorded what was done during the consultations. This varied per GP as well as per patient and per consultation. Table 2 provides examples of data extracted from electronic medical records of three patients. There are 2 examples of brief records (≤10 words), 2 examples of medium-length records (11–30 words) and 2 examples of long records (≥31 words).The table also illustrates how these were categorised according to the GP guideline categories. An overview of the strategies the GPs used in the 1035 consultations is provided in Table 3. The most common diagnostic strategies were physical examination (24.5% of consultations, range among GPs 7.0–66.7%) and additional investigations within the GP practice (14.6%, range 0–50%). Of the additional investigations, laboratory tests such as various blood, urine and feces tests were done most frequently (11.1%, range 0–37.5%). Symptom exploration was recorded in 3.5% of the consultations (range 0–20.0%) and found among 40% of the GPs. Having administered the recommended symptom checklist enquiring about distress, depression, anxiety and somatisation symptoms, the 4-Dimensional Symptom Questionnaire (4DSQ) [22], was only recorded once (0.1%).

**Table 2:** Examples of categorisation of data extracted from electronic medical records

Patient	Length of record	Information extracted from medical records	Categorised as
113	≤10 words	Had a talk. Gave explanation.	*Diagnostic*: None *Therapeutic*: - Education and explanation - Discussing progress - Other: “talk”
171	≤10 words	Physical examination, referral to neurologist	*Diagnostic*: - Physical examination *Therapeutic*: - Referral to secondary care (unclear for diagnostics or treatment): neurologist
115	11–30 words	Explained that I don’t know whether a scan is indicated, but that due to the long duration of complaints we can ask for the orthopedist’s opinion: referral	*Diagnostic*: - Diagnostic referral *Therapeutic*: - Education and explanation
123	11–30 words	Stop tramal, start fentanyl patch, and follow up appointment after 2 weeks, is allergic to diclofenac, developed a rash, fentanyl patch 12 mcg/hr. 5 pieces	*Diagnostic*: None *Therapeutic*: - Medication adjustment: discontinuation - Prescribed medication: opioids - Follow-up appointment
158	≥31 words	Carried out physical examination. Exploration. Does not feel reassured despite good lab results and echo abdomen. Will go to exercise therapist and an optometrist for visual test. Will return in a month for an evaluation. If there is insufficient improvement, referral to a psychiatrist. In my opinion no indication of physical cause. Patient will also fill in a diary with symptoms (because complaints are very inconsistent). Explanation when to return sooner.	*Diagnostic*: - Exploration of symptoms - Physical examination - Discussing test results *Therapeutic*: - Education and explanation - Symptom diary - Discussing progress - Follow-up appointment
165	≥31 words	Gave explanation: No somatic problem, no reason to be extra vigilant with normal heartbeat. Talked about the option to talk to the behaviour specialist, is going to do this. Will go to physiotherapist to learn not to focus on his normal heartbeat. Wants to go there as well because wants to hear from a professional whether everything is OK during a workout, prefers not to start a long treatment program (psychosomatic physiotherapy?)	*Diagnostic*: None *Therapeutic*: - Education and explanation - Referral within primary care: other GP consulting another health professional: other

**Table 3:** Overview of management strategies

Management strategies	n of consultations ^a^ (%) ^b^	n of patients
Diagnostic strategies		
Exploration of symptoms	36 (3.5)	22
Physical examination	254 (24.5)	67
Additional investigations within GP practice	151 (14.6)	61
Laboratory tests	115 (11.1)	57
ECG	17 (1.6)	14
X-ray	22 (2.1)	16
Echography	7 (0.7)	7
Other	7 (0.7)	7
Diagnostic referral	34 (3.3)	27
Discussing test results	62 (6.0)	39
Therapeutic strategies		
Shared problem definition	4 (0.4)	4
Education and explanation	116 (11.2)	46
Advice	112 (10.8)	45
Lifestyle/dietary advice	53 (5.1)	31
Physical exercise advice	52 (5.0)	28
Other advice	17 (1.7)	13
Symptom diary	12 (1.2)	10
Shared plan for symptom management	48 (4.6)	28
Setting up a time contingent plan	1 (0.1)	1
Discussing/giving advice about medication	97 (9.4)	35
Medication	255 (24.6)	65
Over the counter medication (OTC)	69 (6.7)	30
Prescribed medication	201 (19.4)	62
NSAIDs	37 (3.6)	24
Opioids	31 (3.0)	19
Psychopharmacological medication	35 (3.4)	20
Sleeping medication	25 (2.4)	15
Antibiotics	7 (0.7)	6
Other	83 (8.0)	44
Unclear OTC or prescribed medication	6 (0.6)	5
Vitamin pills/injections	121 (11.7)	12
Medication adjustment	71 (6.9)	29
Dose increase	22 (2.1)	15
Dose reduction	18 (1.7)	13
Discontinuation	37 (3.6)	21
Refill prescription	28 (2.7)	11
Referral within primary care	47 (4.5)	31
Physiotherapist	25 (2.4)	18
Mental health nurse practitioner	14 (1.4)	12
Other	9 (0.9)	9
Physiotherapist appointment	27 (2.6)	20
Mental health nurse practitioner appointment	29 (2.8)	8
Referral to secondary care for treatment	26 (2.5)	18
Medical specialist	14 (1.4)	9
Rehabilitation	15 (1.4)	13
Referral to secondary care (unclear for diagnostics or treatment)	46 (4.4)	31
Rheumatologist	11 (1.1)	10
Neurologist	10 (1.0)	9
Gastroenterologist	7 0.7)	6
Internist	6 (0.6)	6
Psychiatrist	1 (0.1)	1
Other	12 (1.1)	12
Referral to a psychologist	5 (0.5)	5
GP consulting another health professional	46 (4.4)	22
Colleague GP	21 (2.0)	6
Secondary care medical specialist	12 (1.2)	9
Other	13 (1.3)	10
Discussing progress	168 (16.2)	52
Follow-up appointment	122 (11.8)	51
Contact if necessary	87 (8.4)	41
Wait and see	97 (9.4)	38
Other	44 (4.3)	26
Unspecified	41 (4.0)	1

^a^Does not add up to 1035 because GPs recorded more than one ICPC codes during one consultation

^b^Does not add up to 100% because GPs recorded more than one ICPC codes during one consultation

The most common treatment strategies were treatment with medication (24.6%, range 0–62.5%), followed by discussing progress (16.2%, range 0–41.5%), scheduling follow-up appointments (11.8%, range 0–33.3%), vitamin pills/injections (11.7%, range 0–36.8%, recorded by less than a quarter of the GPs, mainly in the same patients), providing education and explanation (11.2%, range 0–35.7%) and giving advice (10.8%, range 0–42.3%). Wait and see strategies were also recorded frequently (9.4%, range 0–40.0%). Medication requiring a prescription was prescribed most (at least 19.4% of all treatment strategies, range 0–40.0%). NSAIDs were prescribed most frequently (3.6% of all treatment strategies, range 0–13.5%), followed by psychopharmacological medication (3.4% of all treatment strategies, range 0–20.0%, recorded by almost half of the GPs) and opioids (3.0% of all treatment strategies, range 0–11.5%, also recorded by almost half of the GPs).

Referrals to a psychologist (0.5%, range 0–7.7%, recorded by 17% of the GPs) or a psychiatrist (0.1%), formulation of a shared problem definition (0.4%, range 0–7.7%, recorded by 10% of the GPs) and setting up a time contingent plan (0.1%) were management strategies that were used the least often. When referrals to secondary care were documented, it was often unclear whether the referral was for diagnostic or treatment purposes. Therefore, a category ‘referral to secondary care (unclear for diagnostics or treatment)’ was added. Finally, of the 44 management strategies categorised as ‘other’, GPs coded 28 consultations (2.7%) with ‘talk’, ‘listening ear’ and ‘encouragement’.

### Management strategies across time

We conducted chi-square tests for trend for the largest categories of management strategies (n of consultations ≥75). Over the 5 year time period, there appeared to be significant trends in the course of ‘giving advice’ χ^2^ (1) = 5.73, *p* = 0.017, ‘medication adjustment’ χ^2^ (1) = 11.67, *p* = 0.001, ‘discussing progress’ χ^2^ (1) = 11.31, p = 0.001, ‘scheduling follow-up appointments’ χ^2^ (1) = 10.75, *p* = 0.001 and ‘contact if necessary’ χ^2^ (1) = 4.11, *p* = 0.043. In all the above categories, the proportion of consultations in which the management strategies concerned were provided, increased over time. For all of the above management strategies except ‘contact if necessary’ there was a small decrease in percentage of the management strategy used within consultations after the first year, after which the percentages increased again. For ‘contact if necessary’, the percentage of the consultations increased steadily across time. There were no significant trends over time for the other management strategies.

## Discussion

### Summary

The most frequent management strategies recorded by Dutch GPs included diagnostic procedures such as physical examinations and additional investigations, and therapeutic procedures such as prescribing medication, discussing progress and providing education, explanation and advice. Other strategies that focus more on listening to the patient and involving patients in their own diagnostic and therapeutic process, and decision making, such as ‘exploration of all complaint dimensions’, ‘shared problem definition’, and ‘shared plan for symptom management’ did not seem to be adopted as frequently. These latter management strategies are especially important for MUS patients [23]. Patients were also rarely referred to a psychologist or psychiatrist.

As the symptoms lasted longer, GPs tended to adjust medication more frequently, discuss progress more often, schedule more follow-up appointments and encourage patients more to contact the practice if necessary.

When comparing these strategies to the recommendations in the Dutch guideline, we can conclude that GPs partly used management strategies recommended by the guideline but several essential strategies were missing. This may possibly reflect either the GPs’ or the patients’ reluctance to seek mental health care for complaints that are perceived to be primarily physical. However, another reason could be that GPs in our sample were not sufficiently familiar with the guideline yet, since the guideline was published during the data extraction period. Even if GPs were familiar with the guideline, it may have taken some time to get used to the new approach, and they may not have started applying strategies, such as exploration of all complaint dimensions, with patients whom they had already seen often before.

Our findings could, however, also point to underreporting of these, more ‘conversation-like’ management strategies. Recording behaviour varied widely across GPs, so it is impossible to know whether the strategy was not provided or not recorded. Due to time constraints, GPs may only put the more objective management strategies such as results of physical examinations, additional investigations and medication prescriptions in the medical records.

### Comparison with existing literature

Several studies investigated management of MUS in other countries. A Norwegian study found that the majority of Norwegian GPs offered supportive counselling (64%), followed by prescribing medication (24%) and additional tests or referrals (20%) [8]. In our sample, the rates for prescription of medication (24%) and additional testing or referral (18%) were similar.

An Italian study found that Italian GPs mostly provided reassurance and support, listened to the patient, prescribed medication, ordered further medical tests and provided information [12]. In our study, prescribing medication, doing further testing and providing information were also among the most commonly used strategies, however offering reassurance and support and listening to the patient were recorded less frequently. Although the GPs in our study coded 2,7% of their consultations as ‘having a talk’, ‘listening ear’ and ‘encouragement’, ‘listening to the patient’ was not one of the categories that we used in our classification of management strategies. Also, not all GPs may record their listening behaviour as such in the medical records.

In the dental field adherence to clinical practice guidelines has been found to be up to 72% on average [24]. GPs also do not fully adhere to clinical practice guidelines [25, 26]. GPs report that they are aware of the guidelines, but find it difficult to implement them with all individual patients, as they may feel that the guideline is not always fitting. GPs may therefore prefer to provide personalized care and let the patients have the final say in their treatment [25, 27]. This may also apply to the GPs in our study.

### Strengths and limitations

To the best of our knowledge this is the first study to investigate care for persons with MUS in such detail. A strength of this study is that we used real-world data directly from electronic medical records. We were therefore able to collect detailed information about every MUS consultation. Furthermore, we did not rely on self-report instruments such as surveys or interviews taken from GPs. This possibly led to having gathered more ‘objective’ data, free from various kinds of bias such as recall bias. Another strength is that the choice of categories for classifying management strategies was based on the current Dutch guideline, which provided clear classification options beforehand. At the same time, it must be noted that the guideline is a best practice statement, which is based on meta-analyses of high-quality randomized controlled trials where possible, but is not always the case. As a part of the recorded consultations took place before the guideline was published, a longer period of time is needed to draw firmer conclusions regarding adherence to the guideline.

Another limitation of this study is that by using electronic medical records our data were completely dependent on the registering behaviour of the GPs, which varied in amount of detail and coding. If the GP did not record certain management strategies or symptoms in a consultation, these data were missing. Our data, therefore, do not necessarily reflect what was actually done during the consultation, rather what was done and recorded. A comparative study with recordings of patients with and without USD, and comprehensive recording of all types of management strategies by GPs or videotaped consultations would be helpful in gaining thorough insight in their management and subsequent recording [28, 29].

A final limitation is that it was not possible to decide which consultation was the first consultation in the course of one or more MUS episodes. Because of this, all consultations were analysed as if they are independent. However, this is usually not the case. The policy of the GP can depend on the findings and results from previous consultations.

## Conclusions

This is the first study that explores the primary care data of Dutch patients with MUS. GPs use standardised management strategies for persistent MUS, but seem to prescribe medication possibly more frequently and explore symptoms and refer to mental health care less frequently than desirable. Over time they seemed to adopt more monitoring and supportive management strategies for the same patient. When seeing patients with MUS, GPs should consider exploring cognitive, emotional, behavioural and social dimensions of MUS besides the somatic dimension, involving the patient more in the problem definition and treatment plan, referring to a mental health nurse practitioner within the practice, mental health care outside the practice and thorough recording in medical records.

## Abbreviations

DSM-5: Diagnostic and statistical manual of mental disorders-5
DSM-IV: Diagnostic and statistical manual of mental disorders-IV
ECG: Electrocardiogram
GP: General practitioner
ICPC: International classification of primary care
MHNP: Mental health nurse practitioner
MUS: Medically unexplained symptoms
NSAID: Nonsteroidal anti-inflammatory drugs
OTC: Over-the-counter
RCT: Randomised controlled trial
SCID-I: Structured clinical interview for DSM-IV axis I disorders
SPSS: Statistical package for the social sciences
